# Niche models at inter- and intraspecific levels reveal hierarchical niche differentiation in midwife toads

**DOI:** 10.1038/s41598-020-67992-6

**Published:** 2020-07-02

**Authors:** Eduardo José Rodríguez-Rodríguez, Juan F. Beltrán, Miguel Tejedo, Alfredo G. Nicieza, Diego Llusia, Rafael Márquez, Pedro Aragón

**Affiliations:** 1 Departamento de Zoología, Facultad de Biología, Universidad de Sevilla, Sevilla, Spain; 2 Departamento de Ecología Evolutiva, Estación Biológica de Doñana, CSIC, Sevilla, Spain; 3 Departamento de Biología de Organismos Y Sistemas, Universidad de Oviedo, Oviedo, Spain; 4 Unidad Mixta de Investigación en Biodiversidad (UO-CSIC-PA), Mieres, Spain; 5 Departamento de Ecología, Facultad de Ciencias. Terrestrial Ecology Group, Universidad Autónoma de Madrid, Madrid, Spain; 6 Centro de Investigación en Biodiversidad Y Cambio Global (CIBC-UAM), Universidad Autónoma de Madrid, Madrid, Spain; 7 Fonoteca Zoológica, Departamento de Biodiversidad Y Biología Evolutiva, Museo Nacional de Ciencias Naturales, CSIC, Madrid, Spain; 8 Departamento de Biodiversidad, Ecología Y Evolución, Universidad Complutense de Madrid, Madrid, Spain

**Keywords:** Evolutionary theory, Environmental impact

## Abstract

Variation and population structure play key roles in the speciation process, but adaptive intraspecific genetic variation is commonly ignored when forecasting species niches. Amphibians serve as excellent models for testing how climate and local adaptations shape species distributions due to physiological and dispersal constraints and long generational times. In this study, we analysed the climatic factors driving the evolution of the genus *Alytes* at inter- and intraspecific levels that may limit realized niches. We tested for both differences among the five recognized species and among intraspecific clades for three of the species (*Alytes obstetricans*, *A. cisternasii*, and *A. dickhilleni*). We employed ecological niche models with an ordination approach to perform niche overlap analyses and test hypotheses of niche conservatism or divergence. Our results showed strong differences in the environmental variables affecting species climatic requirements. At the interspecific level, tests of equivalence and similarity revealed that sister species were non-identical in their environmental niches, although they neither were entirely dissimilar. This pattern was also consistent at the intraspecific level, with the exception of *A. cisternasii,* whose clades appeared to have experienced a lower degree of niche divergence than clades of the other species. In conclusion, our results support that *Alytes* toads, examined at both the intra- and interspecific levels, tend to occupy similar, if not identical, climatic environments.

## Introduction

Climatic factors may act as ecological barriers that can determine the distribution of animal and plant species^1^. This may explain why incipient speciation processes often can be inferred from the analysis of patterns of niche divergence^2^. Genetic variation, species spatial structure resulting from landscape barriers to gene flow, and intraspecific evolutionary processes (e.g., local adaptation) are the major drivers of speciation. Therefore, it is not surprising that genetic studies combined with ecological niche models have been proven to be powerful tools to study evolutionary processes and resolve biodiversity conservation problems^3^. In addition, the characterization of environmental niches is essential to understanding species distributions and patterns of biological diversity. Correlative ecological niche modelling^33^ is a common tool used to approach this characterization^3^. However, is the species level the most adequate level for this approach? Smith et al.^4^ suggest that we must consider local adaptations as evolutionary factors affecting niche requirements, and therefore, inclusion of evolutionary relationships below and above the species level should be considered. For this reason, it is informative to compare niche modes across taxa in a separate way, considering species and local genetic lineages. Amongst vertebrates, amphibians are ideal organisms to analyse this question because their physiology is highly constrained by environmental factors^5,55^, and they have low dispersal abilities. This combination of characteristics is expected to promote the evolution of local adaptations to match the spatial complexity of environmental variation^7^.

It is well known that vicariant events can drive divergence by limiting genetic exchange among evolutionary units^8^, but currently, there is evidence that environmental factors can also play a key role in biological diversification^9–11^. Although habitat suitability models and niche similarity comparisons have been previously conducted at interspecific and intraspecific levels for the midwife toads (*Alytes* sp.)*,* these models have been implemented only in *A. obstetricans*^9^. However, other Mediterranean species, such as *A. cisternasii* and *A. dickhilleni*, have not been studied using nested models (inter- and intraspecific climatic niche divergence schemes) despite the fact that their genetic and phylogenetic discontinuities are well documented^12,13^. Furthermore, an interspecific perspective of climatic niches for all the species in the genus is crucial for a better understanding of climatic determinants and the differentiation processes involved.

Recent studies on niche modelling have shown that environmental conditions can drive evolution across geographical ranges and affect patterns of genetic structure^14,9,15^. Moreover, genetic isolation and local adaptation can synergistically influence the fate of species^16^, and biogeographic and vicariant events can drive species differentiation (see Martínez-Solano et al.^17^). However, the environmental factors involved in the maintenance of the current differenciation of species and evolutionary units have not been tested as a whole. Tectonic factors, with the formation of a mountain range in the Gibraltar Strait during the Upper Tortonian stage, played an important role in the speciation of the genus *Alytes* ^18,17^^.^ In fact, the geographic isolation of *A. muletensis* and *A. maurus* might have strongly affected their environmental niches. Finally, recent work suggests that a more complex geological scenario might have affected the evolutionary history of *Alytes* in its southern range, with a Pliocene volcanic archipelago between Cabo de Gata and the eastern Rif coast^19^.

The major aims of this study were (1) to characterize the realized niche differences and environmental factors that promote the differentiation and the observed distribution of *Alytes* species, and (2) to investigate the importance of niche evolution by testing the hypotheses niche conservatism, as the maintenance of ancestral requirements among species with a common ancestor^20^, and niche divergence, as the appearance of divergences among these species^2^. Additionally, we tested whether the observed patterns of niche environmental evolution were consistent at two phylogenetic scales: interspecific and intraspecific (i.e., genetic lineages from Dias et al.^12^; Gonçalves et al.^13^; and Maia-Carvalho et al.^9^). We assumed the existence of differentiation at both inter- and intraspecific levels as a consequence of climatic niche differentiation. In this scheme, intra- and interspecific differentiation may be influenced by climate and geographic barriers throughout genetic variation and structure. Finally, We also aimed to determine whether the processes of niche and phylogenetic evolution were parallel by predicting whether subclades would show a phylogenetic signal.

## Methods

### Organism presence records and environmental data

The genus *Alytes* Wagler 1829 currently contains five living species. *Alytes obstetricans* (Laurenti, 1768) has the broadest geographical range of all the species in this group. *Alytes cisternasii* Boscá 1879 is endemic to the south-western region of the Iberian Peninsula. *Alytes muletensis* (Sanchiz & Adrover 1979) has a narrower distributional range and is endemic to the northern part of Mallorca Island. *Alytes* (*Baleaphryne*) *dickhilleni* Arntzen & García-París 1995 is endemic to the Betic Region on the south-eastern Iberian Peninsula. Finally, *Alytes maurus* Pasteur & Bons 1962, is a species distributed in some regions of the Rif and Middle Atlas of Morocco. Additionally, four recognized subspecies of *A. obstetricans* have been described: *A. o. almogavarii* Arntzen & García-París, 1995, from southern France to the Ebro River; *A. o. obstetricans* (Laurenti 1768), from Western Europe to north of the Iberian Peninsula; *A. o. boscai* Lataste 1879, in central and northern Portugal; and finally *A. o. pertinax* García-París & Martínez-Solano, 2001, on the eastern Iberian Peninsula.

The habitats of these five species show a large degree of differentiation. *A. obstetricans* requires areas with high amounts of precipitation and occupy a wide range of habitats, from mountain ranges to crops^21^. *A. muletensis* is only known in a few localities on northern Mallorca Island^22^, whereas *A. maurus* is restricted to a few localities in the Rif and Middle Atlas Mountains of Morocco and typically occupies humid sites in karst and steep areas^23^. Regarding the two Iberian endemics, *A. dickhilleni* is restricted to the Betic region in southeastern Spain^24^. *A. cisternasii* occupies the central-south-western sector of the Iberian Peninsula, usually between 0 and 700 m^25^, and, in comparison to the other species of this genus, it is associated with a hotter and drier climate^26^.

**Figure 1:**
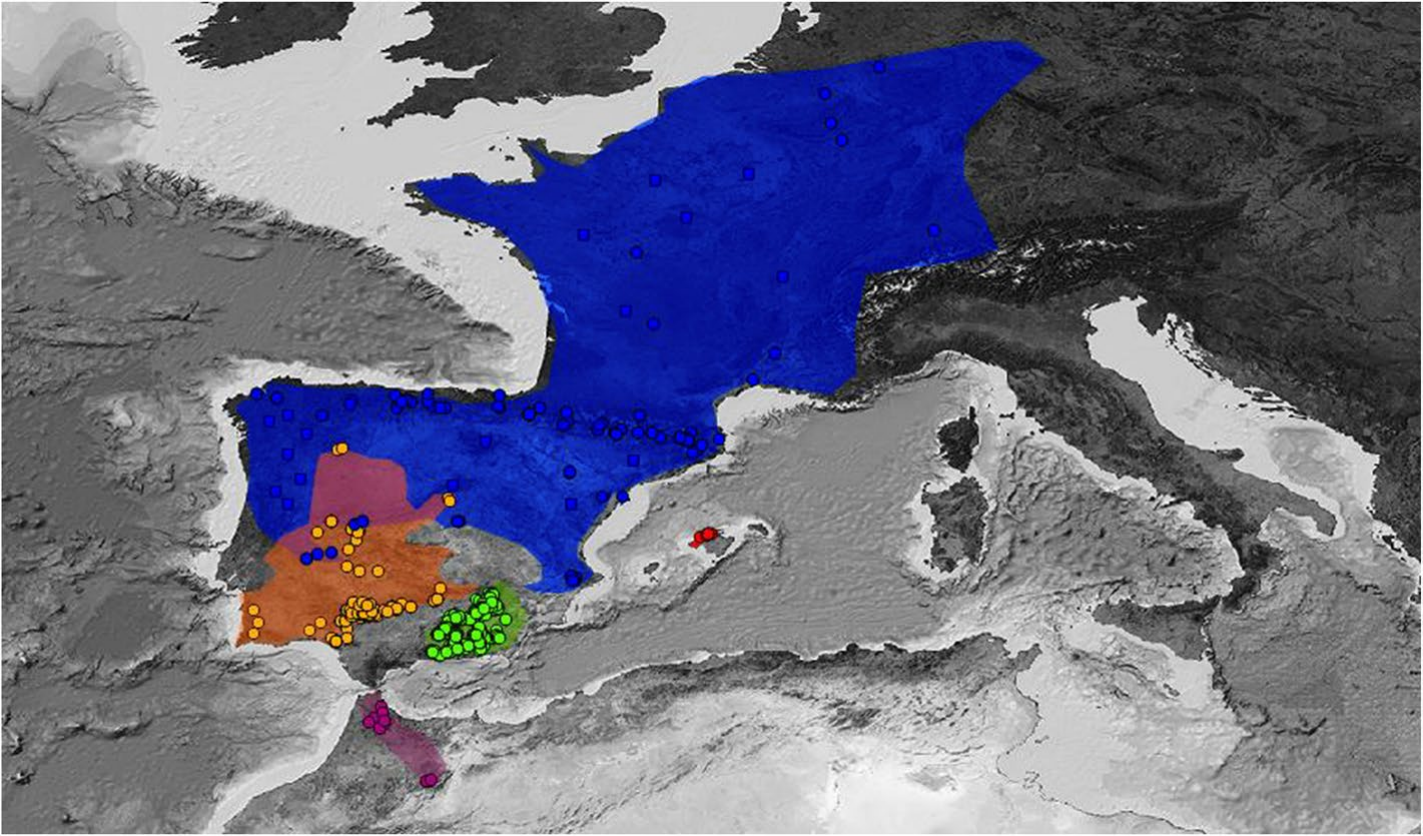
Records of presence included in this study. We considered populations in all the distribution areas, and we only selected populations separated by at least 200 m. A: *Alytes dickhilleni*, B: *Alytes cisternasii*, C: *Alytes maurus*, D*: Alytes obstetricans*, and E: *Alytes muletensis*. Figure created with QGIS Chugiak 2.4.0 (QGIS Development Team. 2018. QGIS Geographic Information System. Open Source Geospatial Foundation Project. https://qgis.osgeo.org). Background map modified from GEBCO Compilation Group (2019) GEBCO 2019 Grid (10.5285/836f016a-33be-6ddc-e053-6c86abc0788e).

We used 676 localities to build ecological niche models. A total of 319 *A. dickhilleni* presence points were surveyed by the authors within all their Andalusian distribution areas. We identified 170 local population locations of *A. cisternasii* (including our own collection data and data from Amphibiaweb^27^), 162 of *A. obstetricans* (our data, Amphibiaweb, and in addition, revised data from www.observation.org), 14 of *A. maurus* (our data, Amphibiaweb and Donaire et al.^23^), and 11 of *A. muletensis* (our data and Amphibiaweb). All populations were separated by at least 200 m. Figure 1 shows the distribution of points selected for all five species.

To establish intraspecific comparisons among *A. cisternasii*, *A. dickhilleni* and *A. obstetricans*, we used presence data for the genetic lineages reported for these species. Presence data were assigned to lineages according to Gonçalves et al. (*A. cisternasii*)^13^, Dias et al. (*A. dickhilleni*)^12^, and Maia-Carvalho et al. (*A. obstetricans*)^9^ (the central-eastern lineage of *A. obstetricans* was excluded because of the low number of presence points). Some local populations could not be assigned to any of the reported lineages, as these locations are in contact zones our outside the areas surveyed in the cited studies, and therefore, these were removed from the data set. We excluded 42 locations of *A. dickhilleni* and 6 of *A. cisternasii*. For the geographically restricted *A. muletensis* and *A. maurus*, the low number of records and lack of adequate genetic lineage information precluded intraspecific analysis. Even in a well-designed data survey across all of the range of a species, model outputs are sensitive to sampling bias^28^. To reduce this bias, we used a sub-sample representing 25% of the populations of each species and lineages included in the study (random selection by MaxEnt). In addition, intraspecific figures show the presence points that were selected for each lineage and then used in the intraspecific correlative model.

We considered climatic and topographic factors to explain the realized distributions and niches of the five *Alytes* species. Climatic variables were obtained from WorldClim version 2 database at 30 s^29^, and topographic data were obtained from SRTM (https://www2.jpl.nasa.gov/srtm/dataprelimdescriptions.html). The study area was fixed to the distribution limits of the *Alytes* sp. Genus, and in order to avoid a high correlation and redundancy among the predictors, we performed pairwise Pearson correlations, and for *r* > 0.6, the variable with lower biological relevance was excluded. The final data set included five climatic variables: mean diurnal range, isothermality, minimum temperature of the coldest month, mean temperature of the driest quarter, and precipitation of the wettest month.

### Niche model analyses

We performed two different modelling approaches: ecological niche models (ENMs) and an ordination technique^30^. First, of the different ENMs types, we selected the widely used machine learning method MaxEnt (version 3.4.1) to build the SDMs ^31^. The model was fitted using hinge, product, linear, and quadratic features with a maximum of 10,000 background points, 1,000 replicates, and clamping. Models were fitted by using the area under the ROC curve (AUC and ROC represents “receiver operating characteristic”^31^). We used the Cloglog output format. Although this measure has been extensively used to fit models, its usefulness has been criticized, especially for presence/background models such as MaxEnt. Thus, in addition to AUC, we present the values of its components, sensitivity and/or specificity^32^. To obtain these two indices, the continuous MaxEnt output was transformed into a categorical variable (predicted presence/absence). For this transformation, we applied the threshold of the minimum training presence (the lowest suitability scores associated with the populations of each lineage/species) given in the MaxEnt output sheet. We calculated the specificity from the confusion matrix^33^. This is a conservative and realistic threshold since it may include even small suitability scores whenever the lineages/species are present^34^. In this case, a calculation of sensitivity was not necessary since the applied threshold was fixed at the maximum. In addition, we have followed the Raes and Ter Steege validation method^57^, including 95% I.C AUC values of null models created with random points of the same size of the presences included in our models. ENM AUC values that are higher than their corresponding 95% CI AUC value of the fitted null model, significantly deviate from what would be expected by chance (*p* < 0.05).

The ordination technique approach was applied to perform niche overlap analyses, either between pairs of the five species or between pairs of the lineages within *A. cisternasii*, *A. dickhilleni and A. obstetricans*. We used the tool Ecospat, which incorporates null hypotheses^35^. For these analyses, we performed the following tests: niche equivalency tests (are niches identical?), similarity tests (are niches more similar than expected by chance?), and niche principal component analysis (PCA). As a measurement of the realized niche overlap, we calculated a Schoener’s *D* index through the niche-PCA. This index ranges from 0 to 1 to reflect no overlap to total overlap, respectively ^36^. For both the niche equivalency and similarity tests, we used the argument = ”greater” (overlap greater than expected by chance) to test the conservatism hypothesis and the argument = ”lower” (overlap lower than expected by chance) to test the divergence hypothesis^35^. We performed 1,000 permutations for each analysis. Additionally, we integrated phylogenies (inter- and intraspecific levels) using the age-range correlation function of the Phyloclim package^38^. This function is used to test for phylogenetic signals in patterns of niche overlap. Slopes and intercepts derived from a linear model can be used to characterize speciation mode (allopatric versus sympatric) or niche evolution (conservatism versus flexibility) in the clade^39^.

Regarding the choice of the geographical extent (and as a consequence, environmental background), we used the software QGIS^40^ to compile and process environmental data using the extension point sampling tool^41^. Analyses were conducted using R^42^. The study area for the interspecific analysis with MaxEnt and ECOSPAT was adapted to the total distribution of the genus to allow comparisons. At the intraspecific level, we used the same area in each cluster of lineages (the species ranges).

## Results

**Figure 2:**
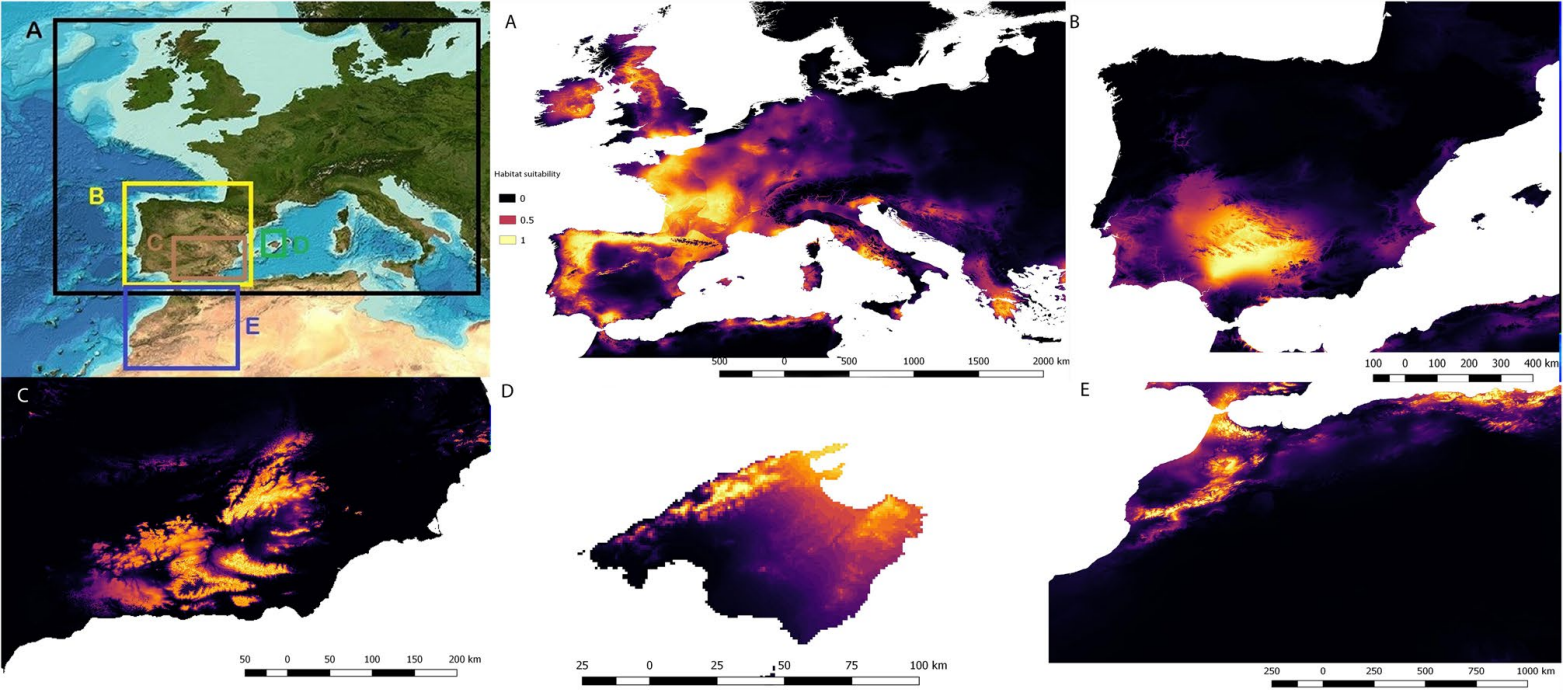
Potential distributions of predicted suitability by Maxent models for *Alytes obstetricans* (**A**), *Alytes cisternasii* (**B**), *Alytes dickhilleni* (**C**), *Alytes muletensis* (**D**) and *Alytes maurus* (**E**). The colour bar is the scale of habitat suitability. Maps created using MaxEnt 3.4.1^31^ and improved with Qgis Chugiak 2.4.0 (QGIS Development Team. 2018. QGIS Geographic Information System. Open Source Geospatial Foundation Project. https://qgis.osgeo.org).

**Figure 3:**
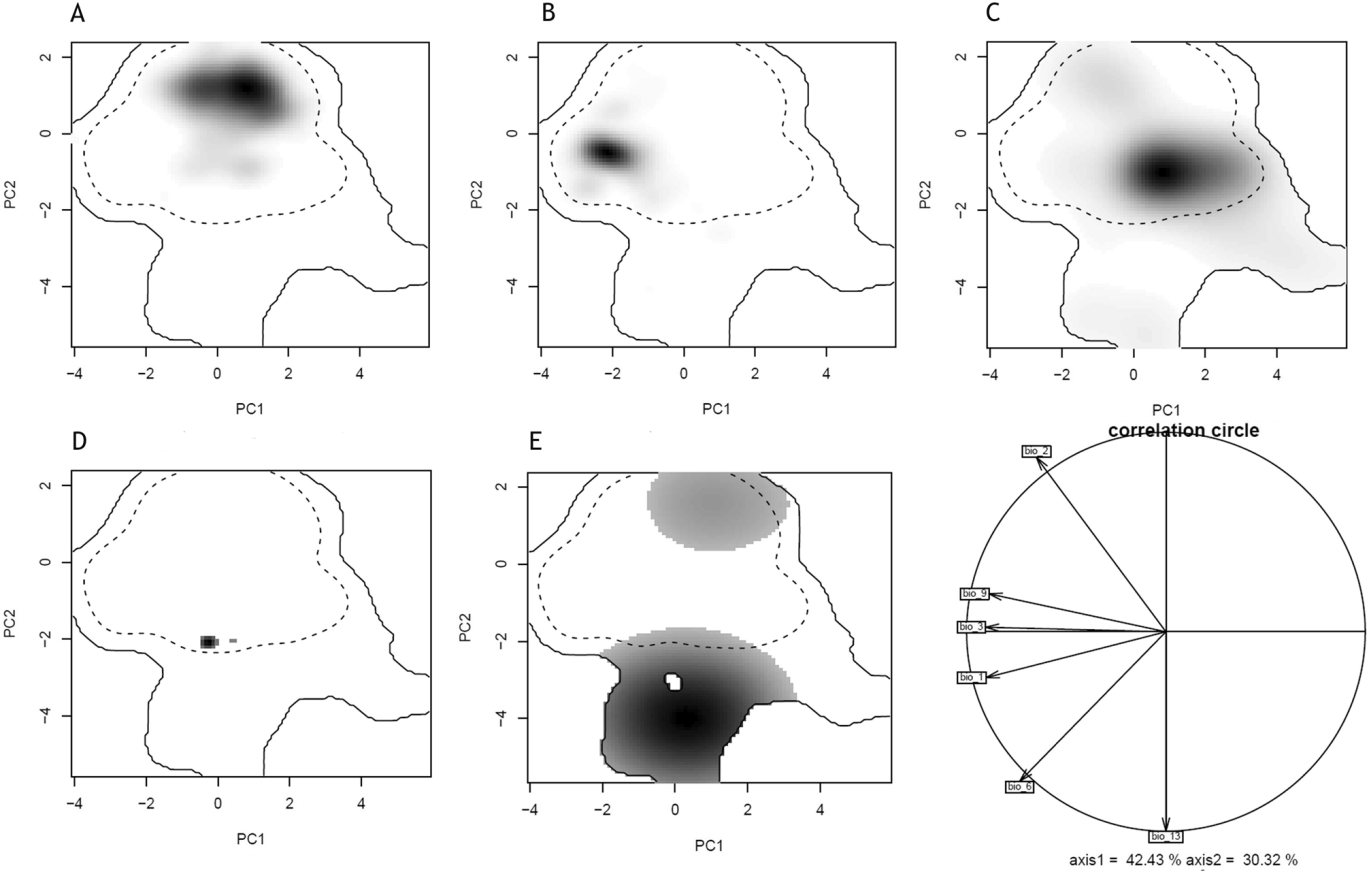
Niches of the five *Alytes* species in the environmental space of the European study area represented along the first principal components (PC) from Ecospat. (**a**) *A. dickhilleni*, (**b**) *A. cisternasii*, (**c**) *A. obstetricans*, (**d**) *A. muletensis* and (**e**) *A. maurus*. (**f**) The contribution of the environmental variables to the two axes of the PC analysis and the percentage of variation explained by the two axes. Variables: annual mean temperature [Bio_1], mean diurnal range [Bio_2], isothermality [Bio_3], minimum temperature of the coldest month [Bio_6], mean temperature of the driest quarter [Bio_9], and precipitation of the wettest month [Bio_13].

At the interspecific level, the Maxent outputs are shown in Fig. 2. The variables with a relatively higher percentage of contribution in the Maxent models were isothermality (62.5%; *A. obstetricans*), mean temperature of the driest quarter (56.3%; *A. cisternasii*), mean temperature of the driest quarter (40.2%; *A. dickhilleni*), precipitation of the wettest month (90.4%; *A. muletensis*) and, finally, precipitation of the wettest month (73.8%; *A. maurus*)*.* The AUC, 95% I.C. AUC of null model and specificity values were 0.90 ± 0.03, 0.86 and 0.65 (*A. obstetricans*); 0.92 ± 0.10, 0.84 and 0.40 (*A. cisternasii*); 0.94 ± 0.02, 0.90 and 0.67 (*A. dickhilleni*), 0.88 ± 0.16, 0.78 and 0.83 (*A. muletensis*), and 0.98 ± 11, 0.92 and 0.95 (*A. maurus*), respectively. The results of the niche overlap (Schoener’s *D*) and similarity and equivalency analyses are shown in Table 1. We found minimal niche overlap with significant *p* values for the equivalency test in the divergence hypothesis, except for the overlap between *A. obstetricans* and *A. maurus*. The magnitude and sign of the variables in the principal component plots of the niche are shown in Fig. 3.

**Table 1:** Schoener’s *D* and *p* values of the five species of *Alytes* (similarity and equivalence tests *p* values for niche conservatism (C) and divergence (D) hypotheses). *Significant *p* values (boldface).

	*A. dickhilleni* versus *A. cisternasii*	*A. dickhilleni* versus *A. obstetricans*	*A. dickhilleni* versus *A. muletensis*	*A. dickhilleni* versus *A. maurus*	*A. cisternasii* versus *A. obstetricans*	*A. cisternasii* versus *A. muletensis*	*A. cisternasii* versus *A. maurus*	*A. obstetricasn* versus *A. muletensis*	*A. obstetrcans* versus *A. maurus*	*A. muletensis* versus *A. maurus*
Schoener’s *D*	0.061	0.106	0	0.013	0.052	0.001	0.018	0.003	0.283	0.014
Equivalency *p* values (C/D)	1/0.009*	1/0.009*	1/0.009*	1/0.009*	1/0.009*	1/0.009*	1/0.009*	1/0.009*	1/0.217	1/0.010*
Similarity *p* values (C/D)	0.48/0.57	0.52/0.46	1/0.54	0.77/0.2	0.544/0.34	0.26/0.66	0.643/0.31	0.18/0.81	0.69/0.79	0.23/0.81

**Figure 4:**
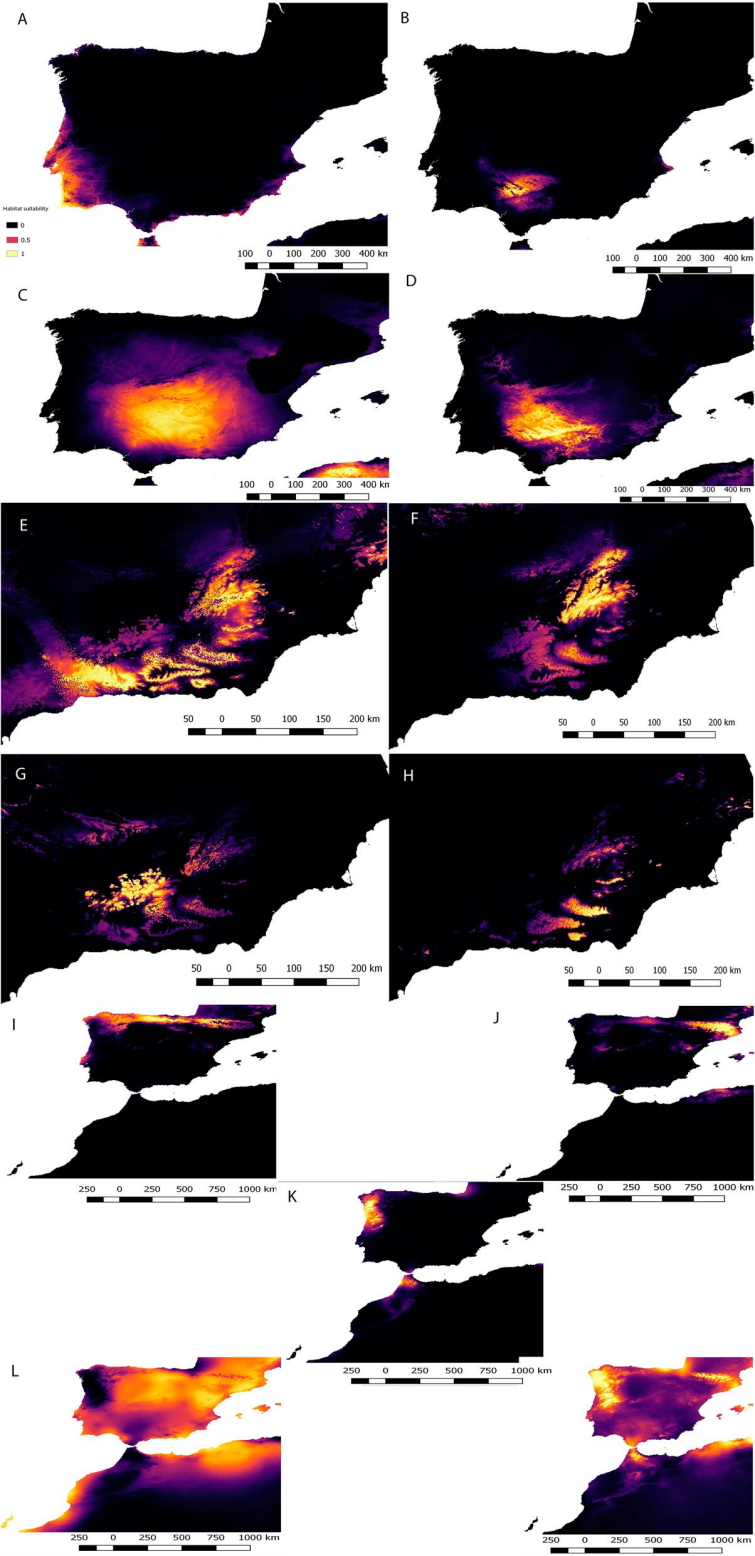
Intraspecific Maxent models of Iberian endemics. *A. cisternasii*. **A**: Western lineage, **B**: southern lineage, **C**: eastern lineage, **D**: and northern lineage; *A. dickhilleni*. **E**: Southern lineage, **F**: northern lineage, **G**: western lineage, and **H**: eastern lineage; *A. obstetricans*. **I**: North-western lineage, **J**: north-eastern lineage, **K**: central-western lineage, **L**: south-western lineage, and **M**: south-eastern lineage. The colour bar is the scale of habitat suitability. Maps created using MaxEnt 3.4.1^31^ and improved with Qgis Chugiak 2.4.0 (QGIS Development Team. 2018. QGIS Geographic Information System. Open Source Geospatial Foundation Project. https://qgis.osgeo.org).

Maxent outputs for intraspecific *A. cisternasii*, *A. dickhilleni* and *A. obstetricans* indicate differences in climatic suitability among lineages. The model outputs are shown in Fig. 4, and the ENM AUC, 95% I.C. AUC of fitted null models and specificity values are shown in Supplementary material 1. The variables with relatively higher contributions in each lineage of *A. cisternasii* were minimum temperature of the coldest month (66.2%) and isothermality (29.7%) for the western lineage, mean diurnal range (47.7%) and precipitation of the wettest month (24.1%) for the southern lineage, mean temperature of the driest quarter (52.3%), isothermality (24.3%) and mean diurnal range (23.4%) for the eastern lineage, and temperature of the driest quarter (45.9%) and mean diurnal range (31.5%) for the northern lineage. For *A. dickhilleni,* the most relevant variables were the mean temperature of the driest quarter (37.8%), precipitation of the wettest month (30%) and isothermality (26.3%) for the southern lineage; annual mean temperature (72.5%) for the northern lineage; precipitation of the wettest month (33.7%), annual mean temperature (29.9%) and isothermality (22.8%) for the western lineage; and annual mean temperature (50.1%) and temperature of the driest quarter (36.6%) for the eastern lineage. Finally, for *A. obstetricans,* the most relevant variables were precipitation of wettest month (90.9%) for the central-western lineage; precipitation of wettest month (36.9%) and annual mean temperature (24%) for the north-eastern lineage, precipitation of wettest month (60.5%) and annual mean temperature (26.7%) for the north-western lineage, mean diurnal range (63.9%) for the south-eastern lineage, and precipitation of wettest month for the south-western lineage.

**Figure 5:**
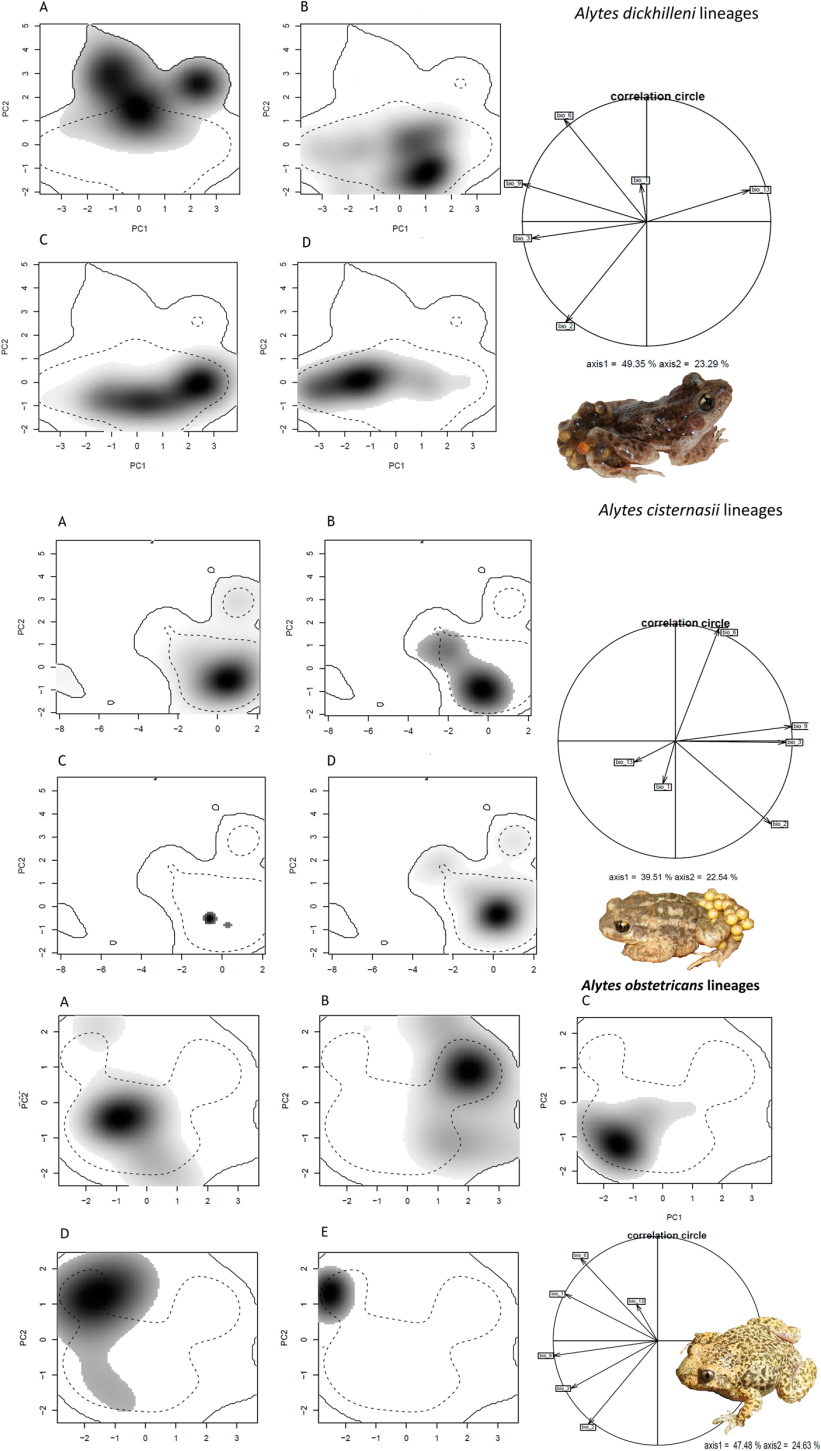
Niches of the phylogenetic lineages of *Alytes dickhilleni* and *Alytes cisternasii* in the environmental space of the Iberian Peninsula and represented along the first principal components axis (PC) from Ecospat under present and climatic change scenarios. For *A. dickhilleni*: (**a**) southern, (**b**) northern, (**c**) eastern, and (**d**) western lineages. For *A. cisternasii*: (**a**) southern, (**b**) eastern, (**c**) western, and (**d**) northern lineages. The contributions of the climatic variables to the two axes of the PC analysis and the percentage of the variation explained by the two axes are also provided. Variables: annual mean temperature [Bio_1], mean diurnal range [Bio_2], isothermality [Bio_3], minimum temperature of the coldest month [Bio_6], mean temperature of the driest quarter [Bio_9], and precipitation of the wettest month [Bio_13]. *A. dickhilleni* and *A. cisternasii* images from the authors and *A. obstetricans* images courtesy of Rafael Carmona González.

The results of niche overlap (Schoener’s *D*) and similarity and equivalency analyses showed low to medium values depending on the pair of lineages compared and the species (see Table 2 for *A. dickhilleni* and *A. cisternasii* and Table 3 for *A. obstetricans*). The south-western lineage of *A. dickhilleni* has lower overlap values than those of the other lineages, and in the equivalency test, we obtained significant results for the divergence hypothesis compared with the niches of the other two lineages (equivalency test, divergence hypothesis, Table 2). There were no significant divergences or convergences between the niches of *A. cisternasii* lineages (Table 2). For *A. obstetricans,* we found significant values in the equivalency test between most lineage comparisons, but we also obtained several significant results for the niche conservatism hypothesis (Table 3). The magnitude and sign of variables in the principal component plots of the niche are provided in Fig. 5. We did not obtain any significance in the age-range correlation test, both at inter- and intraspecific levels (see Supplementary material 2, *p* values of 0.60 [interspecific], 0.28 [*A. cisternasii*], 0.98 [*A. obstetricans*], and 0.64 [*A. dickhilleni*]). In Supplementary material 2 we also provide cluster dendrograms based on niche overlap for both, intraspecific and interspecific levels.

**Table 2:** Schoener’s *D* and *p* values (similarity and specificity for niche conservatism (C) and niche divergence (D) hypothesses of intraspecific lineages for *A. dickhilleni* and *A. cisternasii.* *Significant *p* values (boldface).

*A.dickhilleni*	Southern lineage versus Eastern lineage	Southern lineage versus Nortern lineage	Southern Lineage versus Western lineage	Northern Vs Eastern lineage	Northern lineage versus Western lineage	Eastern lineage versus Western lineage
Schoener’s *D*	0.087	0.032	0.049	0.461	0.342	0.197
Equivalency *p* value (C/D)	1/0.0099*	1/0.0099*	1/0.0099*	1/0.092	1/0.099	1/0.098
Similarity *p* value (C/D)	0.722/0.248	0.811/0.214	0.643/0.294	0.168/0.859	0.138/0.815	0.366/0.849
*A. cisternasii*	Southern lineage versus eastern lineage	Southern lineage versus Western lineage	Southern lineage versus Northern lineage	Eastern lineage versus Western lineage	Eastern lineage versus Northern lineage	Western lineage versus Northern lineage
Schoener’s *D*	0.255	0.010	0.481	0.015	0.279	0.016
Equivalency *p* value (C/D)	0.818/0.363	1/0.091	1/0.091	1/0.090	0.727/0.363	1/0.090
Similarity *p* value (C/D)	0.306/0.683	0.257/0.683	0.267/0.772	0.158/0.881	0.416/0.683	0.257/0.772

**Table 3:** Schoener’s *D* and *p* values (similarity and specificity for niche conservatism (C) and niche divergence (D) hypotheses of intraspecific lineages for *A. obstetricans.* *Significant *p* values (boldface).

*Alytes obstetricans*	Schoener’s *D*	Equivalency *p* value (C/D)	Similarity *p* value(C/D)
North-western versus North-eastern	0.051	1/0.009*	0.665/0.366
North-western versus South-eastern	0.436	1/0.138	0.043*/0.971
North-western versus South-western	0.240	0.831/0.099	0.333/0.633
North-western versus Central-western	0.009	1/0.009*	0.406/0.594
North-eastern versus South- eastern	0.026	1/0.009*	0.673/0.336
North-eastern versus South-western	0.022	1/0.009*	0.643/0.287
North-eastern versus Central-western	0.000	1/0.009*	1/0.386
South-eastern versus South-western	0.165	0.029*/0.0398*	0.415/0.613
South-eastern versus Central-western	0.038	0.009*/0.009*	0.564/0.673
South-western versus Central-eastern	0.194	0.881/0.128	0.137/0.920

## Discussion

Our results showed a clear spatial niche segregation when we examined interspecific niche variation in *Alytes* toads. On the Iberian Peninsula, the distribution outputs of the MaxEnt models fit neatly in some places, although they exhibited a slight overlap in certain areas that coincides with the present sympatric distributional area between *A. cisternasii* and *A. obstetricans*. The pattern derived from niche similarity and equivalence tests revealed that each *Alytes* species occupies a non-identical environmental niche since no significant *p* values were found for the hypothesis of complete niche overlap (equivalence test), but instead, highly significant distinctions occurred for the divergence hypothesis (equivalence test) for any pair-species comparisons with the exception of the *A. obstetricans* versus *A. maurus* contrast. This suggests an evolutionary scenario where niches are less equivalent (identical) than expected by chance in relation to different non-exclusive processes, including local adaptation. In addition, we did not find significant results in the similarity test (conservatism hypothesis), rejecting the hypothesis of retained similarity. We also did not find significance in the case of the divergence hypothesis for the similarity test, indicating no greater than expected divergence. However, rejecting the retained similarity, determining that the divergence was not greater than expected was already relatively divergent. *A. cisternasii* has been described as the most phylogenetically distant group within the genus *Alytes*^17^. This is compatible with an evolutionary scenario where the complex formed by *A. obstetricans*, *A. maurus*, *A. muletensis*, and *A. dickhilleni* share a more recent natural history and, consequently, they could also share similar environmental/climatic requirements to a high degree. However, our results did not support this prediction, thus suggesting that the niches of these species evolved in a complex scenario, creating a wide diversity of adaptations. *A. maurus* and *A. obstericans* presented the widest climatic range (see Fig. 2). In comparison to the other species (with the exception of *A. maurus*), *A. obstetricans* inhabits colder areas with higher precipitation. *A. cisternasii* occupies warmer areas with relatively high precipitation (at least in the wettest period); *A. maurus* faces a wider range of temperatures and a high rainfall amount but with the widest variable range. It is important to remark that our models shows two separate areas in the ecological niche models (Ecospat) of *A.maurus*, this being possibly this an artefact due to the lack of knowledge about the distribution or alternatively the isolation of the two extant populations^56^. In turn, in comparison to *A. cisternasii* and *A. muletensis*, *A. dickhilleni* is present in colder and drier areas. Finally, *A. muletensis* occupies warm and dry areas, although the distribution of this species was much wider in the past^44^; in addition, the current distribution may be restricted to highly isolated populations due to non-climatic factors such as intense predation pressure by the introduced water snake *Natrix maura*^43^. This scenario may bias the output of our correlative model, which relies on distributional and climatic factors. A process to determine the robustness of our approach could be to implement mechanistic physiologically based models and to examine the congruence of both approaches^55^.

When examining the degree of environmental niche evolution at the intraspecific level, we found contrasting patterns across and within species. The four clades of *A. cisternasii* tended to exhibit slight niche differentiation differences between the four genetically distinct clades, although correlative models showed different predicted distributions of the four lineages, with the western lineage being the most different, as it is associated with more humid conditions, than those the three other lineages that form a complex with reduced niche differentiation, in agreement with the phylogenetic tree proposed by Gonçalves et al.^13^. However, a contrasting pattern was found in *A. dickhilleni,* whose southern lineage exhibited significant non-equivalence with respect to the other three lineages, reflecting a more separated evolutionary history for this clade. Interestingly, this southern lineage differentiation in climatic requirements, characterized by a reduced diurnal range and mild winters (temperature of the coldest month) whereas the other three lineages exhibited differentiation in their climatic niches, fits well with the observed pattern of genetic divergence, by which the southern clade formed a sister group to a complex containing the other three lineages^12^. Finally, clades of the widespread *Alytes obstetricans* exhibited the highest diversity in the pattern of climatic niche evolution with high niche conservatism. The two southernmost lineages between the north-western and south-eastern lineages (in the similarity test) suggest a diminished importance of niche divergence for the intraspecific lineages described for this species. In addition, we did not find support for the existence of phylogenetic signals in the age-range correlation tests (both at interspecific and intraspecific levels). This allowed us to consider the flexibility in the niche evolution hypothesis as opposed to niche conservatism reported for other groups^36^^.^

Climatic conditions are important factors influencing both the inter- and intraspecific evolution of *Alytes* and consequently its ecological niche segregation. The evolutionary history of this genus seems to be the result of a combination of vicariant factors influenced by both landscape and geographic factors^17^. Our results for *Alytes* reinforce the idea that intraspecific variability can be one of the major drivers of biodiversity^46^. Our results also match the conclusions of Maia-Carvalho et al.^9^ about the ongoing processes of differentiation in *A. obstetricans* but provide a more general, wider view of the generation of diversity. Thus, vicariant and geographic barriers explain the current patterns (inter- and intraspecific) of diversification; environmental and geographical factors can act synergistically to drive differentiation at multiple scales. The intraspecific differences may be explained by the most commonly accepted evolutionary alternatives: (A) niche conservatism that may be the consequences of natural selection^47^. We observed a lower intraspecific niche divergence in *A. cisternasii* than in *A. dickhilleni* and, at the same time, a higher phylogenetic influence (*p* value lower). This may possibly be induced by its specialized thermophilic physiology that may constrain uplift dispersion to mountain ranges^48^ (Rodríguez-Rodríguez et al. unpublished data). B) Other selective sources affect relatively lower dispersal ability due to ecomorphological constraints. *A. cisternasii* is the shortest-limbed species in group ^25^, and indirectly, this may be associated with a lower dispersal ability than that of the other *Alytes* species^49,50^.

Regarding the phylogenetic analysis results, we conclude that no phylogenetic signal was detected at either interspecific or intraspecific levels. This fact is congruent with the conclusions the remaining tests that supported the rejection of retained niche similarity (conservatism), suggesting a nom parallelism of phylogenetic inertia and niche evolution.

Our results support a model of hierarchical niche differentiation in midwife toads. This model helps to understand the evolution of this primitive genus of amphibians, but most importantly, this approach has widespread application in conservation biology. First, it demonstrates the need for a modelling strategy based on targets below the species level. Second, it shows that the identification of a relatively reduced series of bioclimatic variables can enable the identification of the most sensitive taxa or lineages^52^. This emphasizes the importance of evolutionary distinctiveness^53^ and the need to connect species range projections with the concept of evolutionarily significant units (ESUs^54^) to prioritize conservation efforts.

## Supplementary information

Supplementary file1

## Data Availability

The datasets generated during and/or analysed during the current study are available from the corresponding author upon reasonable request.
