# Supplementary material for: Niche models at inter- and intraspecific levels reveal hierarchical niche differentiation in midwife toads
Source: Sci Rep. 2020 Jul 2;10:10942. doi: 10.1038/s41598-020-67992-6 (PMC7331615; doi:10.1038/s41598-020-67992-6)
Supplement: Supplementary file 1 — Supplementary file1 [file 41598_2020_67992_MOESM1_ESM.docx]

**Niche models at inter- and intraspecific levels reveal hierarchical niche differentiation in midwife toads**

***Rodríguez-Rodríguez, Eduardo José^1^; Beltrán, Juan F.^1^; Tejedo, Miguel^2^; Nicieza, Alfredo G. ^3,4^; Llusia*^5,6^*, Diego; Márquez*^7^*, Rafael; Aragón, Pedro^8^.***

***^1^Departamento de Zoología. Facultad de Biología. Universidad de Sevilla. Sevilla, Spain.***

***^2^Departamento de Ecología Evolutiva, Estación Biológica de Doñana. CSIC. Sevilla. Spain.***

***^3^Departamento de Biología de Organismos y Sistemas. Universidad de Oviedo, Spain***

***^4^Unidad Mixta de Investigación en Biodiversidad (UO-CSIC-PA). Mieres, Spain***

***^5^Departamento de Ecología. Facultad de Ciencias. Terrestrial Ecology Group. Universidad Autónoma de Madrid. Spain.***

***^6^Centro de Investigación en Biodiversidad y Cambio Global (CIBC-UAM). Universidad Autónoma de Madrid. Spain***

***^7^ Fonoteca Zoológica. Departamento de Biodiversidad y Biología Evolutiva. Museo Nacional de Ciencias Naturales, CSIC, Madrid. Spain.***

***^8^ Departamento de Biodiversidad, Ecología y Evolución. Universidad Complutense de Madrid. Spain.***

**Corresponding author**

**e-mail:** [**edurodrodbio@gmail.com**](mailto:edurodrodbio@gmail.com)

**ORCID: 0000-0002-1170-0788 (Rodríguez-Roríguez EJ),** [**0000-0002-0077-575X**](https://orcid.org/0000-0002-0077-575X) **(Beltrán JF),** [**0000-0003-4183-184X**](https://orcid.org/0000-0003-4183-184X) **(Tejedo M),** [**0000-0003-4062-569X**](https://orcid.org/0000-0003-4062-569X) **(Nicieza A), 0000-0002-2070-860X (Márquez R), 0000-0002-6849-7274 (Aragón P)**

**Supplementary material**

**Supplementary material 1**. AUC and specificity values for each intraspecific lineage model.

| **Species (Lineage)** | **AUC** | **95% IC AUC null models** | **Specificity** |
| --- | --- | --- | --- |
| ***A.cisternasii* (Western)** | 0.99±0.10 | 0.89 | 0.97 |
| ***A.cisternasii***  **(Southern)** | 0.99±0.04 | 0.91 | 0.93 |
| ***A.cisternasii***  **(Eastern)** | 0.93±0.11 | 0.84 | 0.76 |
| ***A.cisternasii***  **(Northern)** | 0.93±0.07 | 0.86 | 0.67 |
| ***A.dickhilleni***  **(Southern)** | 0.95±0.03 | 0.89 | 0.75 |
| ***A.dickhilleni***  **(Northern)** | 0.97±0.06 | 0.90 | 0.81 |
| ***A.dickhilleni***  **(Western)** | 0.97±0.08 | 0.91 | 0.86 |
| ***A.dickhilleni***  **(Eastern)** | 0.99±0.03 | 0.88 | 0.88 |
| ***A.obstetricans***  **(North-eastern)** | 0.93±0.11 | 0.86 | 0.95 |
| ***A.obstetricans***  **(North-western)** | 0.98±0.12 | 0.90 | 0.82 |
| ***A.obstetricans***  **(Central-western)** | 0.99±0.09 | 0.89 | 0.99 |
| ***A.obstetricans***  **(South-eastern)** | 0.84±0.14 | 0.81 | 0.64 |
| ***A.obstetricans***  **(South-western)** | 0.87±0.16 | 0.83 | 0.68 |

**Supplementary material 2. Phyloclim results at intraspecific and interspecific levels; and cluster dendrograms for niche overlap.**

**
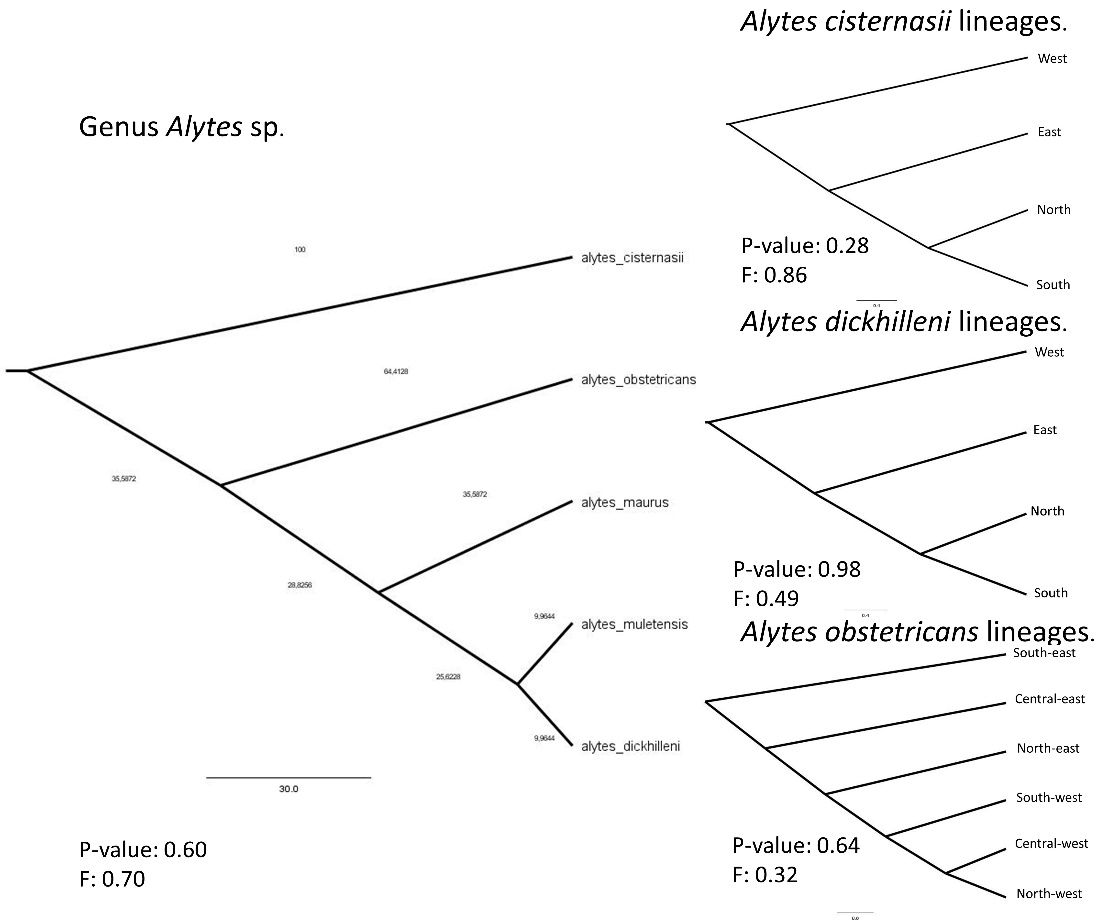
**

**
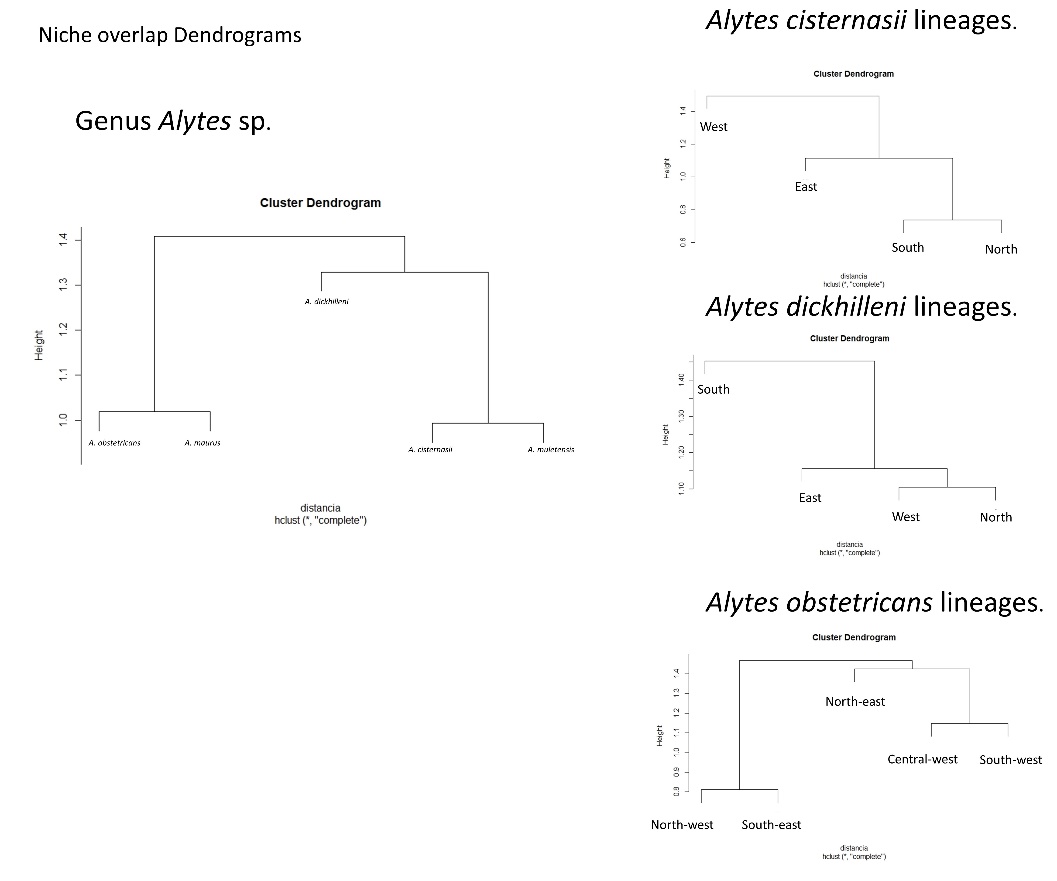
**

**Supplementary material 3.**

##########Script by Rodríguez-Rodríguez, 2020########

######Using Phyloclim niche evolution history (Heibl & Calenge, 2018) ########

#Intalling packages

install.packages(ape)

Install.packages("phyloclim")

#Opening libraries

library("ape")

library("phyloclim")

#Charging phylogeny

a<-file.choose("alytes.nex")

summary(a)

MyTree<-read.nexus(a)

write.nexus(MyTree)

#Plotting phylogeny

plot(MyTree, type = "phylogram", use.edge.length = TRUE,

node.pos = NULL, show.tip.label = TRUE, show.node.label = TRUE,

edge.color = "black", edge.width = 1, edge.lty = 1, font = 3,

cex = par("cex"), adj = NULL, srt = 0, no.margin = FALSE,

root.edge = FALSE, label.offset = 0, underscore = FALSE,

x.lim = NULL, y.lim = NULL, direction = "rightwards",

lab4ut = NULL, tip.color = "black", plot = TRUE,

rotate.tree = 0, open.angle = 0, node.depth = 1,

align.tip.label = FALSE)

#Opening climatic data. In this case I ave imported from excel and named it as CLIM

#Creating niche overlap matrix (Niolap)

niche.overlap(CLIM)

nicheoverlap<-niche.overlap(CLIM)

#Using age range correlation test

age.range.correlation(phy = MyTree, overlap = nicheoverlap, tri = "upper", n= 100)


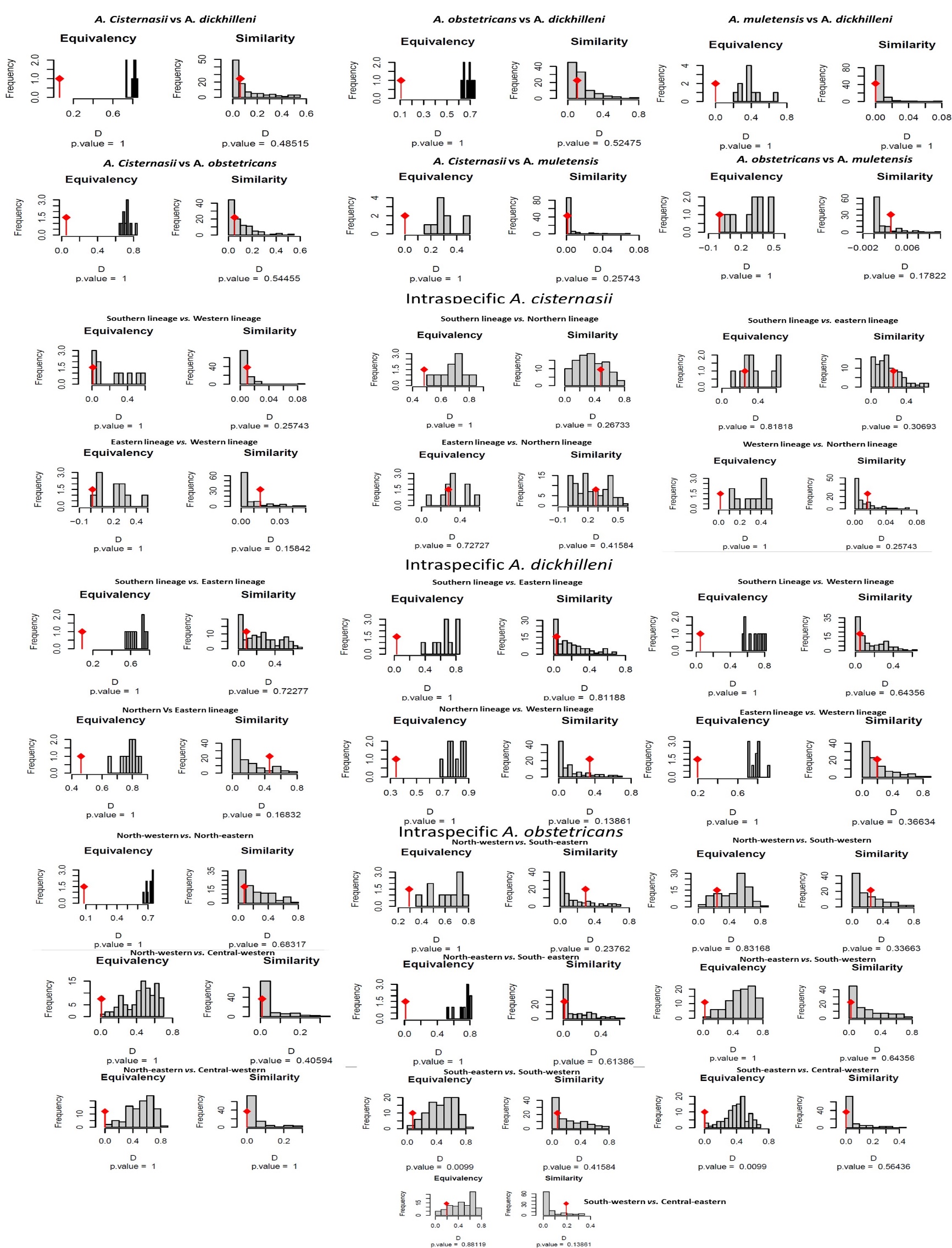
**Supplementary material 4. Histogram outputs for Ecospat**
